# Toward Weight-Inclusive Family-Based Treatment: Clinical Considerations and Opportunities for Change

**DOI:** 10.3390/jcm15218221

**Published:** 2026-10-22

**Authors:** Maria Nicula, Kristen Anderson, Gina Dimitropoulos, Rebecka Peebles

**Affiliations:** 1 Faculty of Social Work, University of Calgary, Calgary, AB T2N 1N4, Canada; 2 Chicago Center for Evidence Based Treatment, Chicago, IL 60602, USA; 3 Monte Nido, Miami, FL 33143, USA

**Keywords:** eating disorders, anorexia nervosa, atypical anorexia nervosa, weight stigma, family-based treatment, weight-inclusive care

## Abstract

Family-based treatment (FBT) is an evidence-based treatment for adolescents with restrictive eating disorders (EDs). However, FBT is delivered within social and healthcare environments where weight stigma and diet culture remain pervasive. This creates a paradox: young people are asked to challenge fears of weight gain, restore weight, and normalize eating, while encountering messages that reinforce thinness, weight loss, and restrictive behaviours. These tensions are particularly salient in FBT, where weight restoration, parent-led renourishment, and discussions of growth and body change are central. In this narrative review, we examine how weight stigma may enter or interfere with FBT principles through the people and processes involved in treatment. Drawing on the existing literature, with additional insights from an author-developed Clinical Teaching Day, we propose practical considerations for delivering weight-inclusive FBT while preserving its core therapeutic functions. These include ongoing clinician self-reflection, weight-inclusive treatment environments, and team alignment around weight-inclusive care, alongside FBT-specific approaches to recognizing illness and activating urgency, establishing treatment targets and supporting weight restoration, supporting parent-led renourishment, navigating weighing and body changes, returning autonomy, and relapse prevention. Weight-inclusive practice need not compete with FBT fidelity; recognizing and addressing weight stigma may instead strengthen clinicians’ ability to achieve FBT’s fundamental aims across the weight spectrum.

## 1. Introduction

Eating disorders (EDs) are serious psychiatric illnesses associated with substantial morbidity [1] and mortality [2,3], with onset commonly occurring during adolescence and young adulthood [4,5,6]. Despite advances in psychological treatments, many young people do not achieve full or sustained recovery [7,8], underscoring the need not only for novel interventions but also for continued refinement of established treatments.

Family-based treatment (FBT) is among the most well-established psychological treatments for adolescents with restrictive EDs [9]. However, even evidence-based treatments are delivered within broader social and healthcare environments that shape how weight, health, eating, and bodies are understood [10]. This is particularly relevant given that approximately one-third of adolescents presenting for FBT have a history of higher weight before their ED began [11,12]. Weight stigma, defined as the social devaluation and negative stereotyping of individuals because of their body weight or size [13,14,15], may represent a potentially under-recognized factor that undermines engagement, treatment processes, or recovery for young people experiencing ED symptoms, particularly those who may need to weight restore to higher growth trajectories to fully recover [16,17].

This creates a unique paradox across the ED care continuum, from emergency departments to primary care settings. Clinicians ask patients to challenge harmful or rigid beliefs or behaviours related to weight, shape, food, and body size while treatment occurs in a society and healthcare system that often reinforces that some bodies are healthier, more desirable, disciplined, or worthy than others. These tensions may become particularly salient in FBT, where weight restoration, parent-led nutritional rehabilitation, and discussions of growth and body change are all central to treatment.

In this review, we examine how weight stigma may enter and interfere with the delivery of FBT and consider how clinicians can respond through weight-inclusive adaptations that preserve the core functions of FBT. The clinical considerations presented here are informed by the existing literature and also draw on a Clinical Teaching Day on weight stigma and FBT developed and delivered by the authors at an international conference.

## 2. The Paradox of Weight Stigma in Eating Disorder Treatment

Taken together, these competing messages create a paradox within ED treatment that manifests in a series of contradictions and potential blind spots.

### 2.1. Weight Loss: Alarming or Celebrated

Sudden, noticeable, or extreme weight loss—or, for some individuals, slowed linear growth—can signal serious illness and prompt urgent intervention in an ED treatment context, yet be encouraged, celebrated, and reinforced elsewhere in healthcare and broader society, particularly for higher-weight patients, even when driven by restrictive eating or other compensatory behaviours [18,19]. Clinicians treating adolescents with atypical anorexia nervosa (AAN), in which all criteria for anorexia nervosa (AN) are met except that weight remains above the threshold considered “significantly low” [20], have described families arriving to treatment confused by this reversal in messaging, having previously been advised to help their child lose weight and now being told that the same child requires renourishment and weight gain [21].

### 2.2. Restriction: Pathological or Overlooked

Restrictive eating and compulsive exercise may raise concern in thinner patients, but be overlooked, normalized, or reinforced in higher-weight patients [18,22]. This is reflected in disparities in ED recognition, diagnosis, referral, and treatment, including longer delays to treatment among higher-weight patients [23,24]. Clinicians have similarly described restriction and weight loss going unnoticed when these behaviours are socially endorsed, as well as greater leniency around return to exercise for some adolescents with AAN [19,21].

### 2.3. Fear of Fatness: Challenged or Reinforced

In ED treatment, patients may be asked to challenge fears of weight gain and fatness or tolerate weight gain while simultaneously navigating weight stigma and anti-fat messages that reinforce those very fears [18,22]. For higher-weight patients, these fears may also reflect previous experiences of weight-based teasing, rejection, or discrimination, leaving clinicians to validate these experiences without reinforcing the ED or undermining necessary weight gain [19,25].

### 2.4. Weight Restoration: Essential or Constrained

Weight restoration toward an individualized weight target is central to many treatments for restrictive EDs [26]. Yet, weight is not synonymous with recovery, which also encompasses psychological, behavioural, and functional outcomes [25,27]. At the same time, determining how much weight restoration is necessary introduces another potential source of weight bias, particularly for higher-weight patients [25]. Patients have described goal weights set close to their current weight, resulting in little or no recommended weight restoration, as well as meal plans being reduced in response to weight gain [28]. Clinicians treating AAN have described uncertainty and disagreement about how far weight restoration should proceed [19,21].

## 3. Family-Based Treatment: An Evidence-Based Treatment for Adolescent Eating Disorders

FBT is a manualized treatment with roots in family therapy approaches developed for adolescents with AN at the Maudsley Hospital in the United Kingdom [29]. It has since been manualized in the United States (first edition in 2001, second in 2013, and third in 2026) [30,31]. FBT has also been adapted for different contexts and populations, including for adolescents with bulimia nervosa (BN) in 2007 [32]. It has since been examined across multiple clinical trials [33,34,35] and has become one of the most strongly-supported outpatient treatments for adolescent AN. Canadian practice guidelines identified FBT as having the strongest evidence among the treatments examined for children and adolescents with AN [9], and family-based approaches specific to EDs are similarly recommended internationally as first-line treatment [36].

FBT is a short-term, three-phase treatment that mobilizes parents and caregivers as an active resource in their child’s recovery, initially focusing on reversing starvation and supporting weight restoration toward remission [31,37]. Two principles are particularly central here. First, FBT is theoretically agnostic regarding the cause of the ED and does not presume underlying family pathology. Parents are explicitly absolved of blame for causing the illness. Second, the ED is externalized from the young person to similarly reduce blame for ED symptoms and support the family to work together toward recovery.

In Phase 1, parents temporarily take primary responsibility for eating and weight restoration. Clinicians create a sense of urgency around the seriousness of the illness while empowering parents to determine how to best renourish their child. Early treatment also includes an in-session family meal, during which the clinician observes and supports the family in overcoming ED-related barriers to eating. Once the adolescent’s weight approaches a point consistent with their historical growth trajectory and conflict around eating has decreased, Phase 2 begins. This phase involves gradually returning responsibility for eating back to the adolescent in an age-appropriate manner. Phase 3 broadens the focus to adolescent development, the adolescent–parent relationship, and preparation for treatment termination.

Despite its strong evidence base, some of the processes through which FBT is delivered may create opportunities for weight stigma to enter or interfere with treatment, particularly for young people and families with diverse bodies and experiences.

## 4. Where Weight Stigma Can Enter or Interfere with FBT

Two aspects of FBT create vulnerabilities to weight stigma: the people involved in treatment and the treatment process itself.

First, the people. FBT brings together the young person, their caregivers, and clinicians, all of whom enter treatment with their own beliefs and experiences related to weight and body size. Parents and clinicians have been exposed to longstanding cultural messages associating thinness with health, success, and self-discipline, particularly for women [38,39]. Parental attitudes and messages about weight and dieting can also be transmitted across generations [40,41]. These messages continue to be reinforced through social media [42], wellness culture [43], and increasing societal attention to and prominence of weight loss and associated medications [44,45,46]. Accordingly, FBT clinicians have identified familial weight stigma and internalized diet culture as barriers to treatment, particularly when trying to support weight restoration among young people with histories of higher weight or experiences of weight-related discrimination [47,48].

Clinicians are also not immune to weight stigma. In one survey of ED professionals, many respondents reported observing colleagues making negative comments or endorsing negative stereotypes about higher-weight patients, or feeling uncomfortable caring for these patients [49]. ED professionals with increased weight bias were more likely to attribute high weight to the patient’s behaviours, were more frustrated or had more negative attitudes about treating higher-weight patients, and assumed that higher-weight patients would have poorer treatment outcomes. Such biases may shape how clinicians recognize and address weight stigma in treatment.

Second, the treatment itself. FBT requires young people, caregivers, and clinicians to repeatedly navigate weight-sensitive tasks and decisions, including interpreting growth trajectories, setting treatment targets, supporting weight restoration, supervising eating and exercise, and returning autonomy to the adolescent. None of these processes are inherently weight-stigmatizing; however, each creates a potential point at which assumptions about weight and health may influence treatment. For example, treatment goal weights may be set too low to reverse weight suppression because of clinicians’ own fears or assumptions about higher weight. Clinicians treating higher-weight adolescents with EDs have described challenges navigating conflicting messages about weight loss, responding to fears of weight gain, uncertainty around treatment targets and weight restoration, and the risk of parental or clinician collusion with the ED, while maintaining fidelity to FBT [19,21]. These challenges highlight the need for practical guidance on delivering the core functions of FBT in ways that recognize and address weight stigma.

## 5. Delivering Weight-Inclusive FBT: Practical Clinical Considerations

Before considering how FBT might be adapted in response to weight stigma, it is important to define weight-inclusive care. Weight-inclusive care refers to an approach that prioritizes health and well-being across the weight spectrum without assuming that body size alone determines one’s health [50]. The clinical considerations that follow draw on the existing literature and were further informed by a four-hour, in-person Clinical Teaching Day (*From Recognition to Action: Addressing Weight Stigma in Family-Based Treatment*) facilitated by the four authors at the 2026 International Conference on Eating Disorders. The author team includes researchers and clinicians with expertise in weight stigma, FBT, adolescent medicine, social work, and clinical education; among the clinical authors are practicing FBT providers, trainers, and supervisors. The workshop included approximately 20–30 participants from varied clinical and leadership backgrounds, including social workers, dietitians, and program directors from several countries. Through didactic teaching, guided reflection, and discussion, participants considered how weight stigma may arise within FBT and identified practical strategies for addressing it in work with families, colleagues, and treatment teams.

Many of these considerations will be familiar to experienced ED clinicians; our intention is not to suggest otherwise, but to highlight points at which weight stigma may nevertheless enter or influence otherwise established clinical practices. Brief hypothetical clinical examples adapted from cases discussed during the Clinical Teaching Day are included throughout this section to illustrate how these considerations may arise and be addressed in practice.

### 5.1. Laying the Groundwork for Weight-Inclusive FBT

#### 5.1.1. Self-Reflection

Delivering weight-inclusive FBT begins with ongoing clinician self-reflection. Clinicians should examine how their own beliefs, assumptions, and experiences related to weight and body size may influence their clinical decision-making. Consistent with FBT’s non-blaming stance, this reflection need not be an exercise in blame or shame. Rather, it requires recognizing that weight-related beliefs are shaped within a broader healthcare context, while taking responsibility for recognizing and addressing how they may influence care. This is not a one-time exercise, but an ongoing practice. Table 1 provides questions to support reflection at personal, clinical, and systemic levels.

#### 5.1.2. Staying Curious and Building Critical Consciousness

Self-reflection should be accompanied by continued learning about weight stigma and critical examination of diet culture and weight-related assumptions. With practice, clinicians may become better able to recognize when weight stigma is entering the treatment space and to respond intentionally when it does. Developing this awareness is an ongoing process rather than a competency that is achieved once.

#### 5.1.3. Creating Conditions for Weight-Inclusive Care

Weight-inclusive FBT also requires attention to the environment and team in which it is delivered. Although many clinicians working in the ED space will be familiar with principles for reducing weight stigma in clinical environments, stigmatizing cues and messages may persist within broader healthcare settings, particularly when care spans teams, departments, or clinical contexts. Physical spaces should accommodate diverse bodies, including through appropriately sized seating and equipment. Teams should also consider less obvious environmental cues that may reinforce weight stigma; for example, analog scales that visibly position a young person toward the higher end of a weight range or weight-loss messaging and materials, such as advertisements for weight-loss studies in hallways or waiting rooms within the ED treatment setting.

A cohesive, consistent approach across the treatment team is equally important. Weight-stigmatizing comments, body talk, dieting discussions, or humour among staff can reinforce weight-centric norms even when they occur outside of interactions with patients and families. Teams can instead create opportunities to discuss weight stigma explicitly, reflect together on clinical encounters (such as by using the questions in Table 1), and normalize respectfully discussing instances in which weight bias may have influenced care.

Workshop discussions particularly highlighted the challenges created by conflicting messages across providers. Where multiple clinicians are involved (within or from outside of the ED treatment setting), clinicians should also anticipate potentially conflicting messages about weight, eating, or weight loss. When these arise, clinicians can acknowledge the confusion they may create for families (such as by validating the confusion) and, where appropriate, work with these other clinicians to align the recommendations. Developing shared weight-inclusive language, protocols, and resources accessible to the whole team can further support consistency and reduce the likelihood that a family receives substantially different messages depending on who provides their care.

**Clinical example.** A young person receiving FBT is advised by the ED team to prioritize nutritional rehabilitation, while another healthcare provider continues to recommend “healthy eating”, portion control, and avoiding “too much” weight gain. The clinician acknowledges the family’s understandable confusion, clarifies how ED recovery recommendations differ from general weight-focused health messaging, and, where appropriate, collaborates with the other provider to align recommendations.

### 5.2. Guiding Principle for Weight-Inclusive FBT

Across all phases of FBT, weight-inclusive care should mean not withholding, delaying, or diluting evidence-based ED treatment based on weight, body size, or shape. Rather, clinicians are encouraged to preserve the core functions of FBT across the weight spectrum, while remaining attentive to where weight stigma may influence how those functions are interpreted or delivered. Drawing on the distinction between an intervention’s core functions and the forms through which they are achieved [51], clinicians can adapt the form when needed while preserving the therapeutic function of FBT. Adaptations should address barriers to recovery rather than make assumptions about what a young person in a particular body should need or can tolerate.

The considerations below are organized according to the phases of FBT. Within each phase, we highlight situations that may signal that weight stigma is influencing treatment (watch for), alongside strategies that clinicians may consider in response (consider).

### 5.3. Phase 1: Supporting Renourishment and Weight Restoration

Weight stigma may be particularly salient during Phase 1, when treatment emphasizes parental responsibility for renourishment, establishing treatment targets, and supporting weight restoration.

#### 5.3.1. Recognizing Illness and Activating Urgency

FBT begins by establishing the seriousness of the ED and creating a sense of urgency around treatment, a process that may be undermined when body size influences perceptions of illness severity. In workshop discussions, this arose particularly in relation to assumptions about what an ED “looks like” when a young person is not at a low weight.

**Watch for:**

- Descriptions about a young person as having “not much to gain” or assumptions that treatment duration will be shorter or less intensive because of their current body size.
- Reduced urgency around restriction or weight loss when a young person is at a higher weight.
- Reluctance to recommend or fully implement FBT because a higher-weight young person is perceived as less likely to require or benefit from treatment, compared to lower-weight patients.
- Greater attention to current body size than to restrictive behaviours, weight trajectory, medical or psychological indicators, growth, or functional impairment.
- Previous or concurrent messages from healthcare providers encouraging weight loss despite restrictive ED symptoms.

**Consider:**

- Most critically, using the patient’s individual growth curve when determining a target weight, and using visual representations of these trajectories to help families understand the medical, psychological, and social consequences and burden of EDs, restriction, and malnutrition across the weight spectrum [6,52,53,54].
- Centering restrictive behaviours, weight trajectory, growth, medical and psychological indicators, and functional impairment rather than current body size or appearance [1,16,52,53,55].
- Applying the same sense of ED seriousness and urgency consistently across body sizes.
- Addressing previous or conflicting messages about weight loss and emphasizing that EDs can be serious across the weight spectrum [55].
- Examining whether assumptions about what an ED “looks like” are influencing diagnosis, treatment recommendations, or the urgency communicated to the family.

The diagnosis of AAN may itself provide an opportunity to examine how weight status shapes the recognition and interpretation of restrictive ED pathology [18,56,57]. Individuals with AAN meet the criteria for AN, except that, despite significant weight loss, their weight remains above the threshold considered “significantly low”. Clinicians must be mindful that the term ‘atypical’ or the absence of a low-weight presentation should not be interpreted by young people, families, or clinicians as indicating that the illness is less serious or requires less intensive treatment.

**Clinical example.** A 16-year-old with AAN has continued to lose weight and become increasingly rigid around eating while waiting for treatment. Because their current body size does not fit the family’s expectations of what an ED “looks like”, their caregivers struggle to understand the seriousness of the illness. The clinician uses the young person’s growth trajectory, degree of weight loss, medical and psychological indicators, and functional impairment to communicate illness severity and build urgency needed for treatment.

#### 5.3.2. Establishing Treatment Targets and Supporting Weight Restoration

Establishing an appropriate treatment target is particularly consequential in FBT because progress toward weight restoration informs the transition from parent-led renourishment in Phase 1 toward the gradual return of responsibility in Phase 2. Workshop discussions further highlighted how discomfort with continued weight gain may influence perceptions of when restoration is sufficient or whether weight should be maintained.

**Watch for:**

- Discomfort from young people, caregivers, or clinicians when an individualized treatment target involves substantial additional weight gain.
- Concerns that weight restoration will cause the young person to become “too large” or “overshoot”.
- Pressure to select the lower end of a treatment range or reduce a target to alleviate distress about weight gain.
- Disagreement within the treatment team about treatment targets that appears to be influenced by the young person’s current or anticipated body size.
- Reduced urgency around continued weight restoration once the young person reaches a weight or BMI conventionally considered “normal”.

**Consider:**

- Establishing treatment targets using the patient’s individualized growth history and developmental trajectory [16,58], rather than relying solely on generic formulas or population-based approaches, such as median BMI [59].
- Avoiding lowering a treatment target primarily to alleviate discomfort about weight gain.
- Recognizing that treatment targets may need to change as children and adolescents continue to grow and develop [58].
- Taking weight suppression (the difference between current and highest premorbid weight) into account when assessing the extent of restoration required, given that higher weight suppression is positively associated with increased ED symptomatology and reduced remission rates [60,61].
- Avoiding intentionally slowing restoration or limiting necessary weight gain because of a young person’s current or previous higher weight.
- Continuing to assess physiological and psychological recovery rather than assuming restoration is complete because the young person has entered a “normal” weight range [55].
- Reframing treatment goals around nourishment, growth, functioning, and recovery rather than achieving or avoiding a particular body size.
- Modeling confidence in the body changes required for recovery rather than inadvertently communicating that the weight gain feared by the ED should also be feared by the family and the treatment team.

#### 5.3.3. Supporting Parents Through Renourishment

Parent-led renourishment may also surface caregivers’ own beliefs and anxieties about food, weight, and body size. Workshop discussions highlighted how concerns about “healthy eating” or providing the “right” foods may inadvertently increase rigidity or interfere with adequate renourishment.

**Watch for:**

- Caregiver discomfort with the amount of food required during renourishment.
- Moralized descriptions of food as “good”, “bad”, “healthy”, or “unhealthy”.
- Concern that, because the young person is eating relatively little, the foods they do eat should be particularly “healthy”.
- Reliance on food guides, balanced-plate models, or other rules in ways that increase rigidity or interfere with adequate renourishment.
- Jokes or comments during meals suggesting that the young person eating the amount required for recovery would make someone else, such as themselves, “fat”.
- Less willingness to provide adequate nourishment because the young person is already in, or is approaching, a higher-weight body.

**Consider:**

- Examining whether language about food inadvertently reinforces weight-based assumptions about health, eating, or body size, including through the use of moralized food categories.
- Explaining how rigid “healthy eating” rules—whether reinforced through schools, public health messaging, or self-imposed—may reinforce restriction and interfere with nutritional rehabilitation [62,63,64].
- Prioritizing adequacy and flexibility over “perfect” nutrition during renourishment
- Individualizing nutritional requirements rather than assuming that a higher-weight young person requires less energy for recovery.
- Introducing feared foods and supporting dietary flexibility as clinically appropriate.
- Interrupting weight- or body-focused comments when they arise and redirect the family toward the immediate task of supporting recovery.

#### 5.3.4. Navigating Weighing, Body Changes, and Fear of Weight Gain

Weight restoration inevitably involves changes in weight and body size, which may elicit distress for both young people and caregivers. Fear of weight gain may be driven by the ED and addressed as part of treatment, but may also reflect very real experiences or fears of weight-based stigma and discrimination [18]. Workshop discussions highlighted how weighing and the communication of weight information may intersect with both sources of distress, requiring particular care in how these concerns are understood and addressed.

**Watch for:**

- Significant distress surrounding weighing or disclosure of weight.
- Increasing fear, shame, or resistance as the young person’s body changes.
- Caregiver distress about continued weight gain.
- Weight-based teasing, discrimination, or stigmatizing experiences contributing to fears about restoration.
- Attempts to reassure the young person by promising that they will not gain “too much” weight or become “fat”, which may inadvertently validate fears of weight gain and reinforce the idea that a higher body weight is an undesirable outcome.
- Pressure from the young person, family, or treatment team to end treatment prematurely because of concerns about continued weight gain or increasing body size.

**Consider:**

- Discussing the purpose and process of weighing and considering how weight information is communicated within FBT and across treatment.
- Where weighing itself is producing substantial distress or treatment interference, consider whether modifications to how weighing is handled are clinically appropriate.
- Continuing to externalize ED-driven fears of weight gain, while avoiding the assumption that every concern about living in a larger body is “just the ED talking”.
- Validating real or anticipated experiences of weight stigma without reinforcing avoidance of weight restoration [16].
- Providing psychoeducation to the families about body diversity, diet culture, and weight stigma.
- Helping young people distinguish the harms of weight stigma from the value or acceptability of their own bodies.
- Modeling confidence and distress tolerance around the body changes required for recovery.

There is currently no clear evidence that open or blind weighing is superior across ED treatment contexts. Open weighing is often grounded in exposure-based principles, providing opportunities to challenge weight-related beliefs and build tolerance of uncertainty [65] as well as some evidence of reduced fear of knowing weight [66]. Blind weighing may reduce immediate weighing-related anxiety and weight preoccupation and may be preferred by some patients, particularly earlier in treatment [67]. Given mixed findings regarding their relative acceptability and effectiveness [68,69,70], clinicians may consider treatment stage, patient preference, and the function of weighing within treatment when determining how weight information is communicated.

**Clinical example.** A 15-year-old with AAN becomes highly distressed during open weighing and remains preoccupied with the number for several days afterward. Rather than abandoning the therapeutic purpose of weighing, the clinician explores what drives the distress, including ED-related fears and experiences of weight stigma, and considers whether the way weight information is communicated should be modified while continuing to support necessary weight restoration.

### 5.4. Phase 2: Returning Autonomy Without Returning to Diet Culture

As responsibility for eating gradually returns to the young person, clinicians and caregivers should attend not only to whether responsibility is being transferred, but also to the beliefs and behaviours accompanying that autonomy. Although many of the warning signs for ED recurrence will be familiar to clinicians, weight stigma and diet culture may normalize or provide socially acceptable language for restrictive or compensatory behaviours, potentially making their re-emergence more difficult to recognize [16,71].

**Watch for:**

- “Healthy eating”, dieting, or new food rules becoming a route back to restriction or weight suppression.
- Exercise being reintroduced primarily for weight control, compensation, or body change.
- Restriction emerging in response to discomfort with the young person’s restored body or exposure to weight-stigmatizing messages.
- Caregivers becoming more permissive of restrictive behaviours because the young person has “weight restored” or is now in a higher-weight body.
- Continued growth or weight gain being interpreted as evidence that nutritional support is no longer necessary.

**Consider:**

- Returning autonomy according to recovery progress and developmental readiness, rather than body size.
- Helping young people and caregivers distinguish flexible, recovery-supportive health behaviours from ED behaviours framed as “health” or “wellness”.
- Continuing to monitor restriction, compensatory exercise, and other ED behaviours regardless of current weight, and return to Phase 1 and greater parental support when needed.
- Explicitly discussing how diet culture and weight stigma may influence eating, exercise, and body-related decisions as autonomy increases.
- Supporting caregivers in tolerating continued growth and body change without returning to weight-focused monitoring or control.

### 5.5. Phase 3 and Beyond: Supporting Recovery in a Weight-Stigmatizing World

As treatment shifts toward adolescent development, relapse prevention, and life beyond FBT and an ED, young people may increasingly encounter environments in which dieting, weight loss, and body dissatisfaction are normalized or encouraged. Weight-inclusive relapse prevention therefore includes building the young person’s capacity to recognize and navigate these messages without returning to ED behaviours [16]. Navigating one’s body size and weight trajectory in this context can be particularly challenging for young people in larger bodies and should not be framed as requiring straightforward or complete body acceptance. Developing greater comfort with, or acceptance of, one’s body may be an ongoing process that extends well beyond FBT and, for some, across the lifespan.

**Prepare the young person for:**

- Weight- or body-related comments from peers, family members, or others.
- Dieting, wellness, and weight-loss content on social media.
- Healthcare encounters in which intentional weight loss is recommended.
- Ongoing changes in body size associated with growth and development.
- Restriction, dieting, or compulsive exercise re-emerging under the language of health.

**Help the young person build capacity to:**

- Recognize weight stigma and critically evaluate and challenge diet-culture messages.
- Respond to, challenge, or disengage from weight-based comments.
- Communicate their ED history and recovery needs in healthcare settings when appropriate.
- Distinguish flexible, health-supportive behaviours from renewed dieting, restriction, compulsive exercise, or other weight-control behaviours that may signal relapse.
- Seek additional support or return to treatment when ED behaviours begin to re-emerge.

Importantly, the above considerations are relevant across the weight spectrum, even though weight stigma may be especially visible or consequential for youth in larger bodies. Bodies and societal ideals change over time, and young people internalize messages about weight and bodies modeled around them. Consistently modeling weight-inclusive language and attitudes can help all young people develop more flexible ways of understanding their bodies and navigating future body changes and weight-related messages. Weight-inclusive care should therefore be understood as good ED care, rather than an adaptation reserved for those in larger bodies.

## 6. Proposed Future Directions

Future work should move from individual clinician adaptation toward standardized guidance for addressing weight stigma while maintaining fidelity to FBT [21], ideally developed in collaboration with FBT leaders. Research is also needed to understand: how weight stigma and diet culture influence engagement and outcomes in FBT [17,48]; whether adaptations such as blind weighing may be appropriate for higher-weight patients whose fear of weight gain is shaped by weight-based discrimination without compromising treatment effectiveness, building on emerging research in this area [72]; and what weight-inclusive FBT should and can look like from the perspectives of both clinicians and higher-weight young people.

## 7. Conclusions

FBT remains one of the strongest evidence-supported treatments available for young people with restrictive EDs. Weight-inclusive practice need not compete with FBT fidelity. Rather, recognizing and responding to weight stigma may instead help clinicians better achieve FBT’s fundamental therapeutic aims. Moving forward, we should therefore ask not whether the FBT itself should change, but where its delivery must become more responsive to the weight-stigmatizing environments in which young people and their families live.

## Figures and Tables

**Table 1:** Reflective questions for recognizing weight stigma in FBT.

Level	Reflective Questions
Personal	What experiences and messages have shaped how I think about bodies, weight, and health? What fears or assumptions arise for me around weight gain, whether in patients or even myself? What thoughts or feelings arise when I imagine my own body changing in ways similar to those I am supporting a patient?
Clinical	How might my own or my team’s assumptions about weight and health influence the care we provide? How do I react when a patient’s body size does not match my expectations of what an eating disorder “looks like”? When might I have unintentionally reinforced weight stigma in my clinical practice?
Systemic	What messages about bodies, health, and worth have been reinforced through my training or healthcare environment? Where do I experience tension between prevailing medical narratives, ED treatment, and weight-inclusive care? How might the systems in which I practice reinforce or hinder weight-inclusive care?
From reflection to action	What do I commit to doing differently to recognize and address weight stigma in my practice?

## Data Availability

No new data were created or analyzed in this study. Data sharing is not applicable to this article.
